# Supplementary material for: Plasmid-mediated phenotypic noise leads to transient antibiotic resistance in bacteria
Source: Nat Commun. 2024 Mar 23;15:2610. doi: 10.1038/s41467-024-45045-0 (PMC10960800; doi:10.1038/s41467-024-45045-0)
Supplement: Supplementary file 1 — Supplementary Information [file 41467_2024_45045_MOESM1_ESM.pdf]

Plasmid-mediated phenotypic noise leads to transient antibiotic resistance in bacteria

SI: SUPPLEMENTARY FIGURES

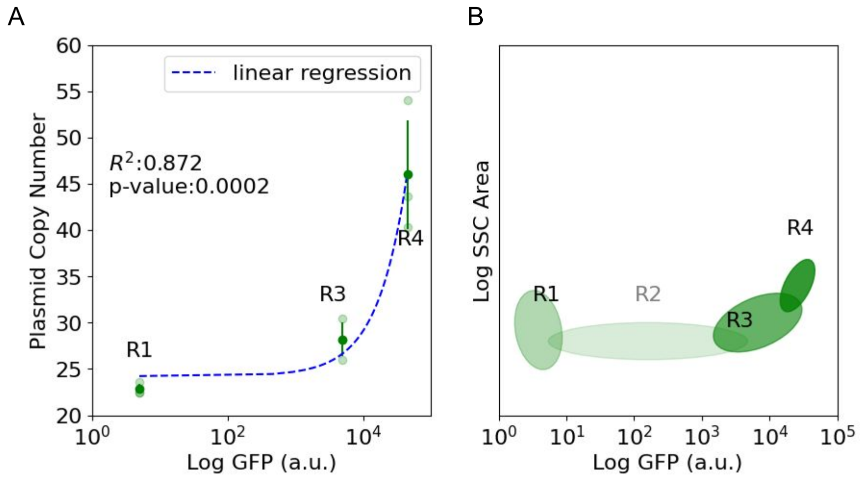

Figure S1. Correlation between PCN and fluorescent intensity. A) Positive correlation between GFP intensity and plasmid copy number estimated using qPCR, for different subpopulations obtained by sorting cells based on their fluorescent intensity. Mean and standard deviation (green) of three technical replicates (shaded green dots). A linear regression is depicted in the dashed blue line. B) Regions used to separate cells based on their fluorescent intensity using a flow cytometer cell sorter. Regions were defined based on standard side scatter area and GFP intensity.

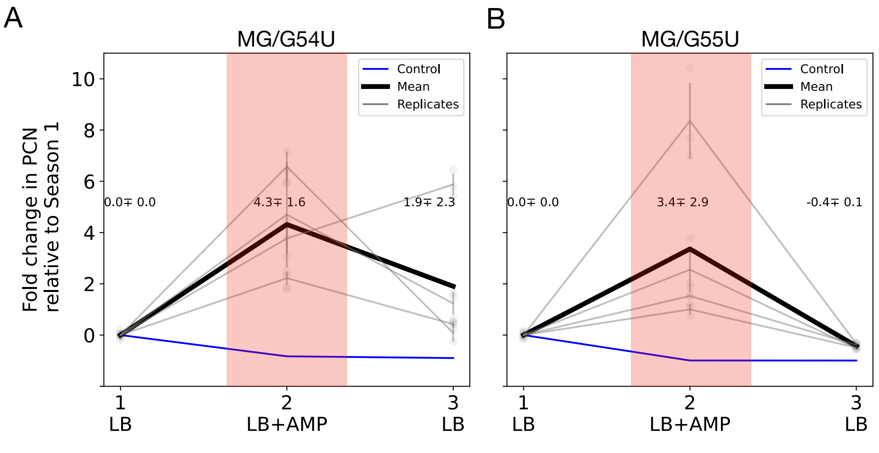

Figure S2. Rapid gene amplification is unstable in high-copy plasmids. A) Fold increase in PCN estimated using qPCR (relative to season 1) for strain MG/G54U in a three-season serial dilution experiment (black line represents the mean over $N = 4$ replicates, in grey). During the second season, a MIC of AMP is deployed (43 mg/ml), selecting for high-copy plasmid cells, therefore increasing five-fold the mean PCN in the population. In the third season, the antibiotic is removed and the mean PCN decrease to the levels exhibited prior to drug exposure. B) Mean PCN for MG/G55U also shows a rapid increase in fluorescence during drug exposure (46 mg/ml) and a rapid decline once the drug is removed.

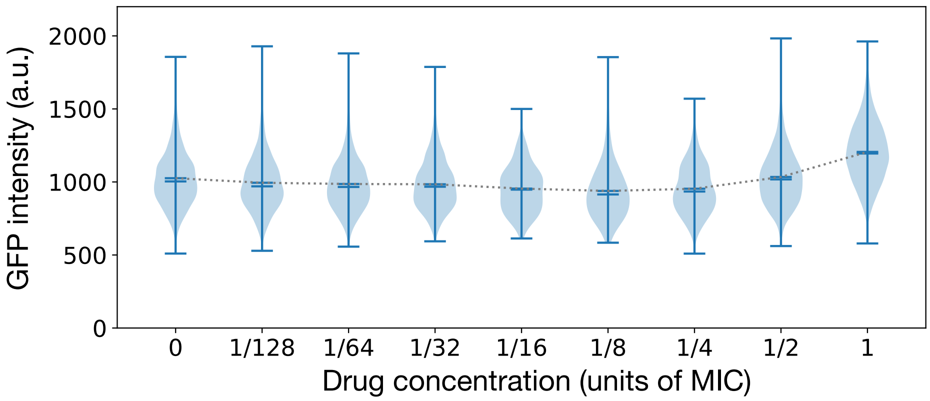

Figure S3. Mean fluorescence of GFP distributions remains constant in populations of MG:GT exposed to increasing concentrations of antibiotic.

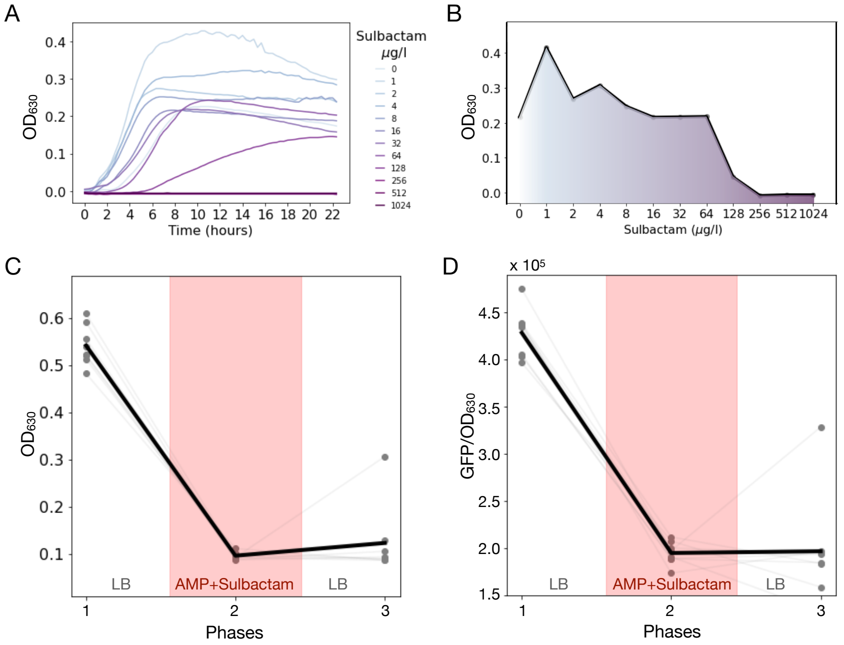

Figure S4. Survival assay with AMP and a β-lactamase inhibitor. A) Growth curves obtained for MG/pBGT populations exposed to 2 mg/ml of AMP and a range of sulbactam concentrations (low sulbactam doses in light blue, high concentrations in purple). B) Final optical density as a function of sulbactam concentration. We consider that bacterial growth is completely suppressed at concentrations higher than $256 \mu g/l$ of sulbactam. C) Optical density (OD_{600}) measured after 12 hours of growth in a 3-season survival assay (season 1: LB; season 2: LB + sulbactam ($256 \mu g/l$) + AMP (2 mg/ml); season 3: LB). Gray lines represent different replicates ($N = 8$), with the mean OD_{600} represented with a black line. Of note, only one replicate exhibited growth after the recovery period. D) Normalized fluorescence intensity of populations exposed to a 3-season serial dilution experiment. Note that supplementing the media with sulbactam reduced the relative fluorescent intensity exhibited by the population during the drug exposure period, in contrast to previous experiments performed in the absence of sulbactam, where we observed an increase in fluorescence during AMP exposure.

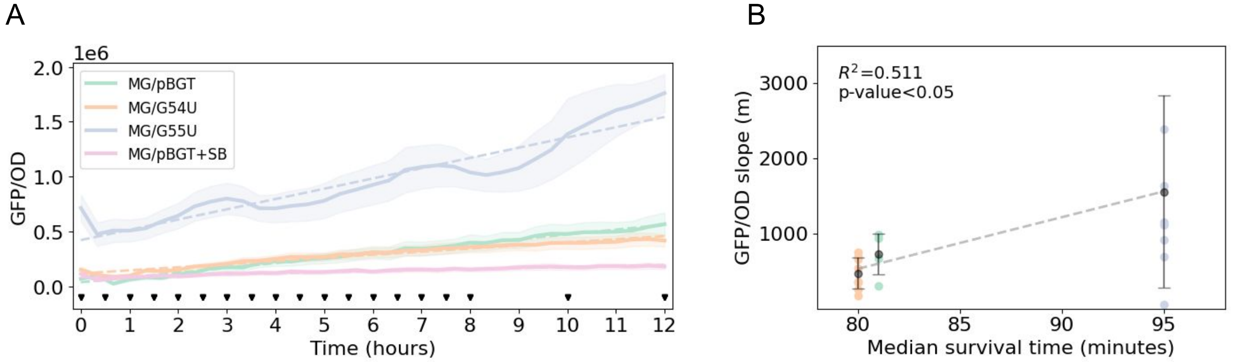

Figure S5. GFP response to antibiotics and survival. A) Per cell GFP fluorescence in different bacterial populations indicates that the increase in fluorescence is associated with the corresponding PCN of each population. Triangles at the bottom illustrate the sampling points used in the survival assay. B) Median survival time (duration of exposure that inhibits the population by 50%) shows a correlation with the observed increase in GFP for populations with different PCN.

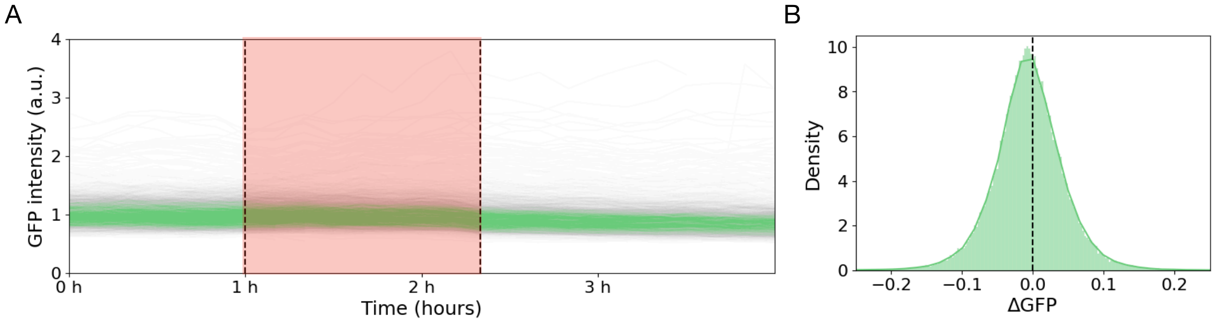

Figure S6. Single-cell time-series of GFP expression. A) GFP fluorescence as a function of time of surviving cells in the MG/pBGT population shows that fluorescent remains constant throughout the experiment. Red area denotes the interval of AMP exposure. B) Difference in GFP between consecutive frames (ΔGFP) during drug exposure. The symmetric distribution suggests there is no increase or decrease in GFP in response to AMP.

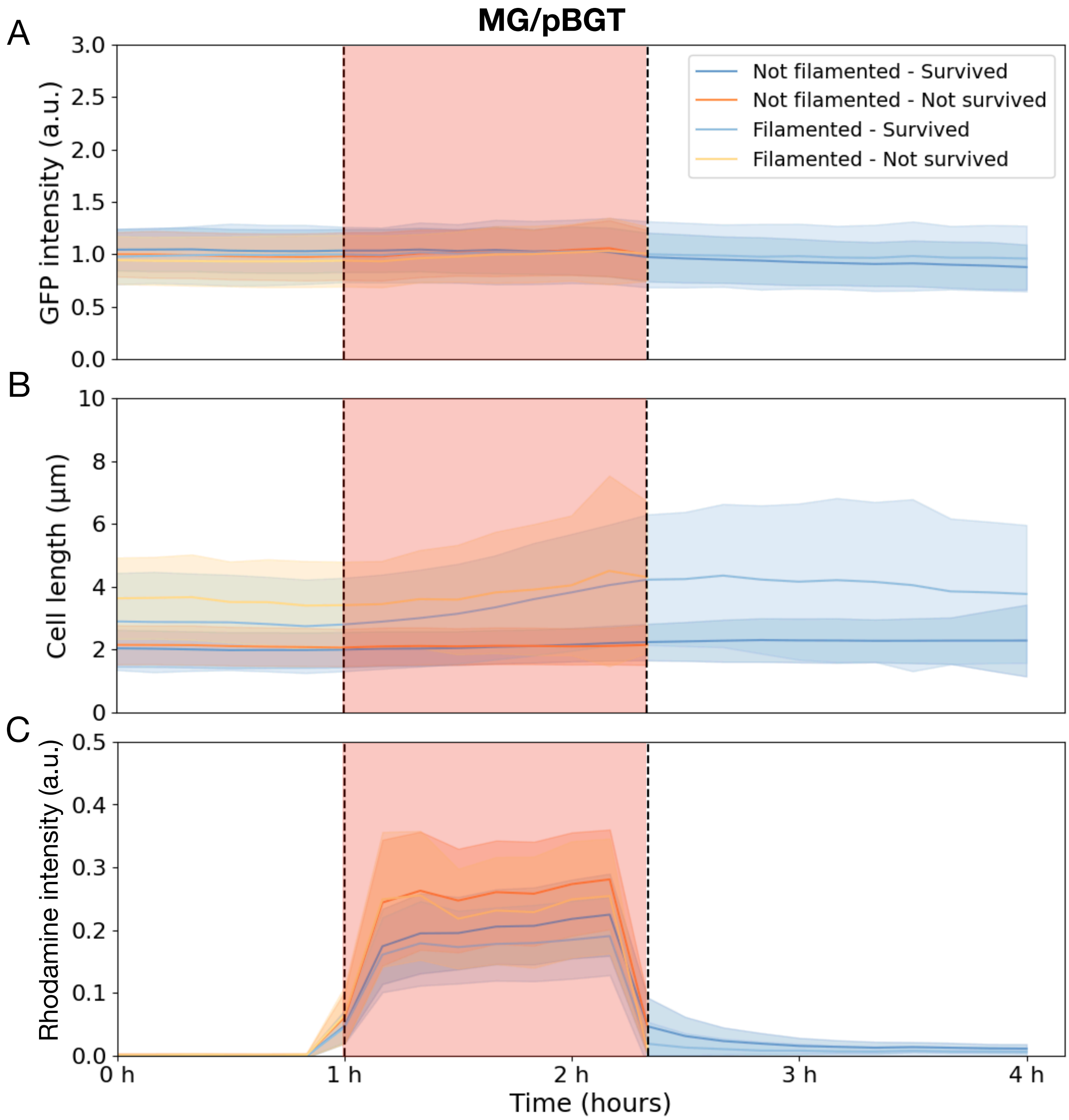

Figure S7. Population-level analysis of microfluidics data for MG/pBGT. A) Normalized GFP intensity as a function of time for different subpopulations: cells that survived (blue) and cells that did not survive (orange). Dark colors represent cells that filaments and light colors represent cells that did not filament. Shaded area representing the standard error over the mean. Red area denotes the duration of antibiotic exposure. B) Cell length as a function of time showing that filamented cells increased cell length during drug exposure. C) Fluorescent intensity in the red channel resulting from a fluorescent dye entering the cells during cell lysis.

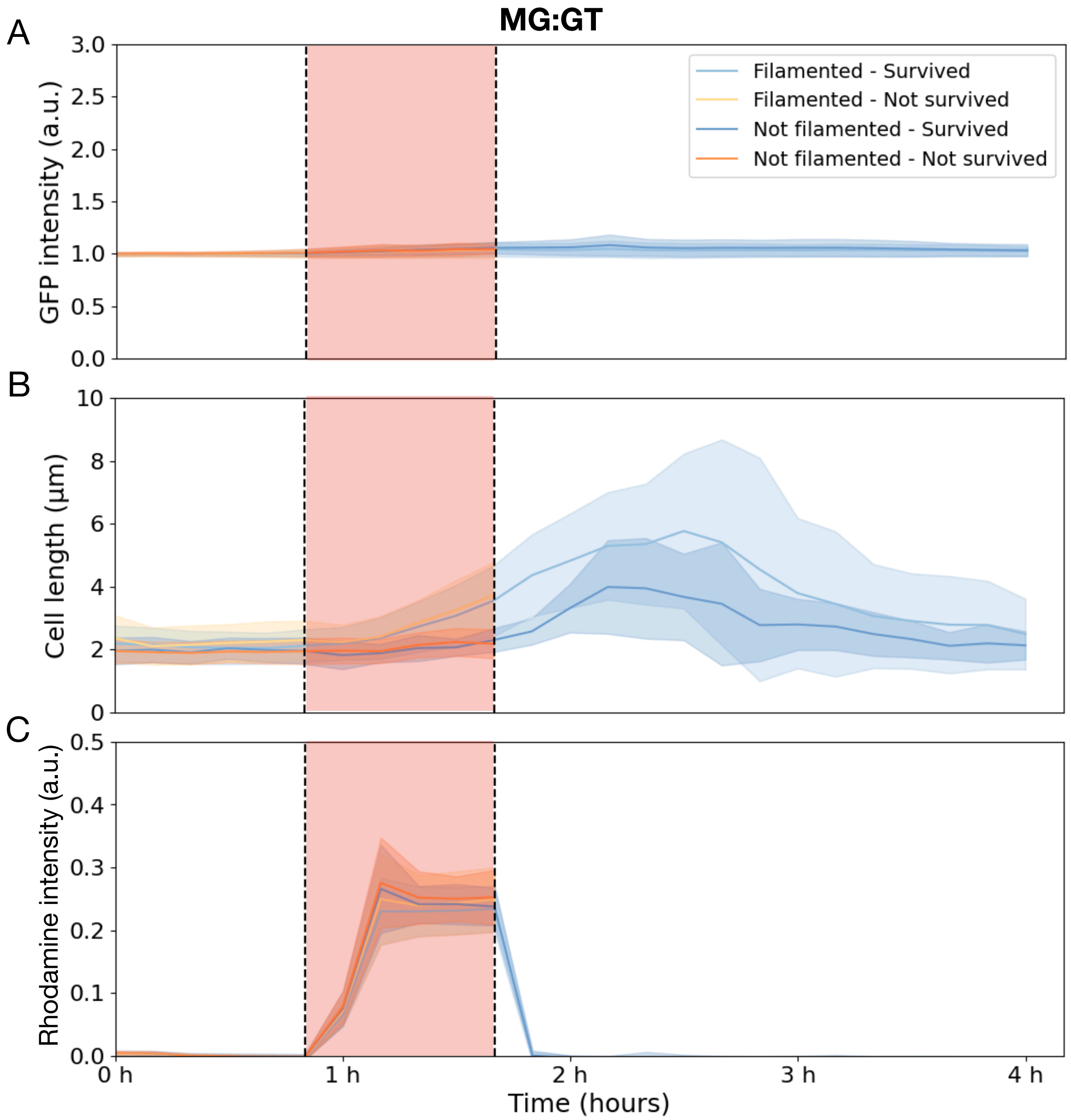

Figure S8. **Population-level analysis of microfluidics data for MG:GT.** A) GFP fluorescent intensity of MG:GT exhibits a stable expression over time, even in the presence of AMP (red area). B) Cell length as a function of time. During drug exposure, most surviving cells increased their cell length. C) Red fluorescent intensity inside each cell. Note a faster increase of fluorescent intensity for the non-surviving populations, consistent with an increase in the concentration of red fluorescent dye inside the cell.

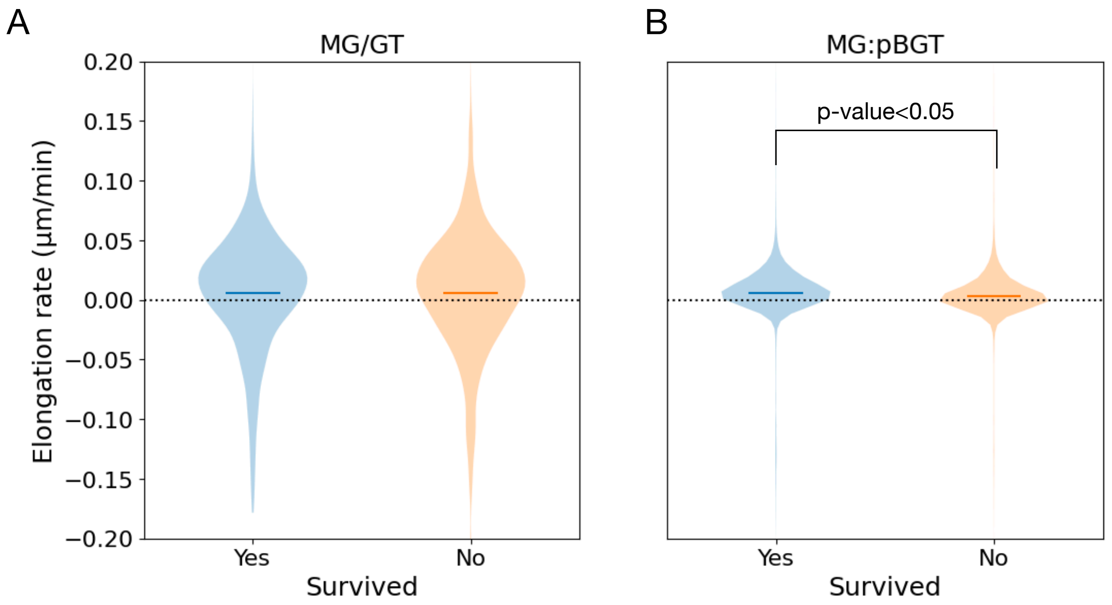

Figure S9. Single-cell elongation rates. Difference in cell length for individual cells in consecutive frames for cells that survived (blue) and did not survive (orange) drug exposure. A) Elongation rate for MG:GT cells. B) Elongation rate for MG/pBGT cells.

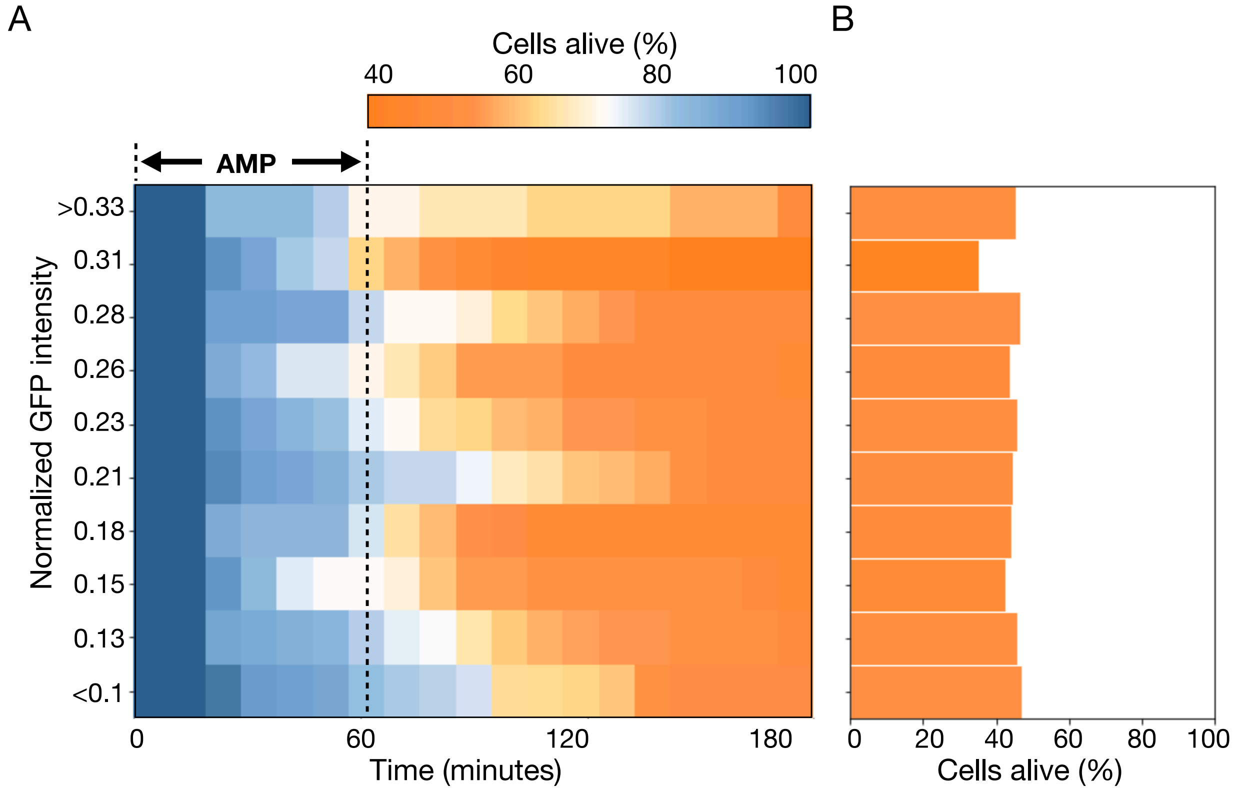

Figure S10. Survival rate and GFP intensity of MG:GT cells. A) Fraction of cells alive are denoted with a color gradient (from orange to blue) at different time-points. Dotted line illustrates the duration of the semi-lethal pulse. B) The percentage of cells that survive treatment remains constant regardless of the initial GFP intensity at the onset of drug exposure.

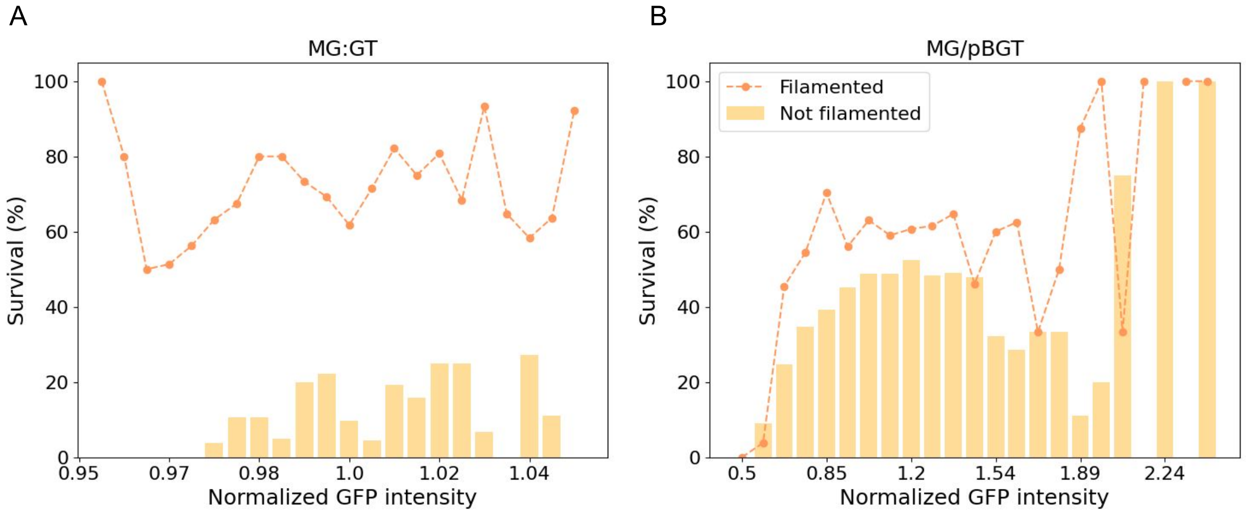

Figure S11. Survival probability of cells with different fluorescent intensities. A) Survival of MG:GT cells that did not produce filaments (bars) for different GFP intensities prior to drug exposure. Cells that filamented (orange lines) exhibited higher survival rates, regardless of their GFP intensity. B) Survival of MG/pBGT cells for different GFP intensities. Note that filamented cells also exhibited a higher survival rate. At higher fluorescent intensities, both filamented and non-filamented cells were able to transiently survive a pulse of a lethal concentration of AMP.

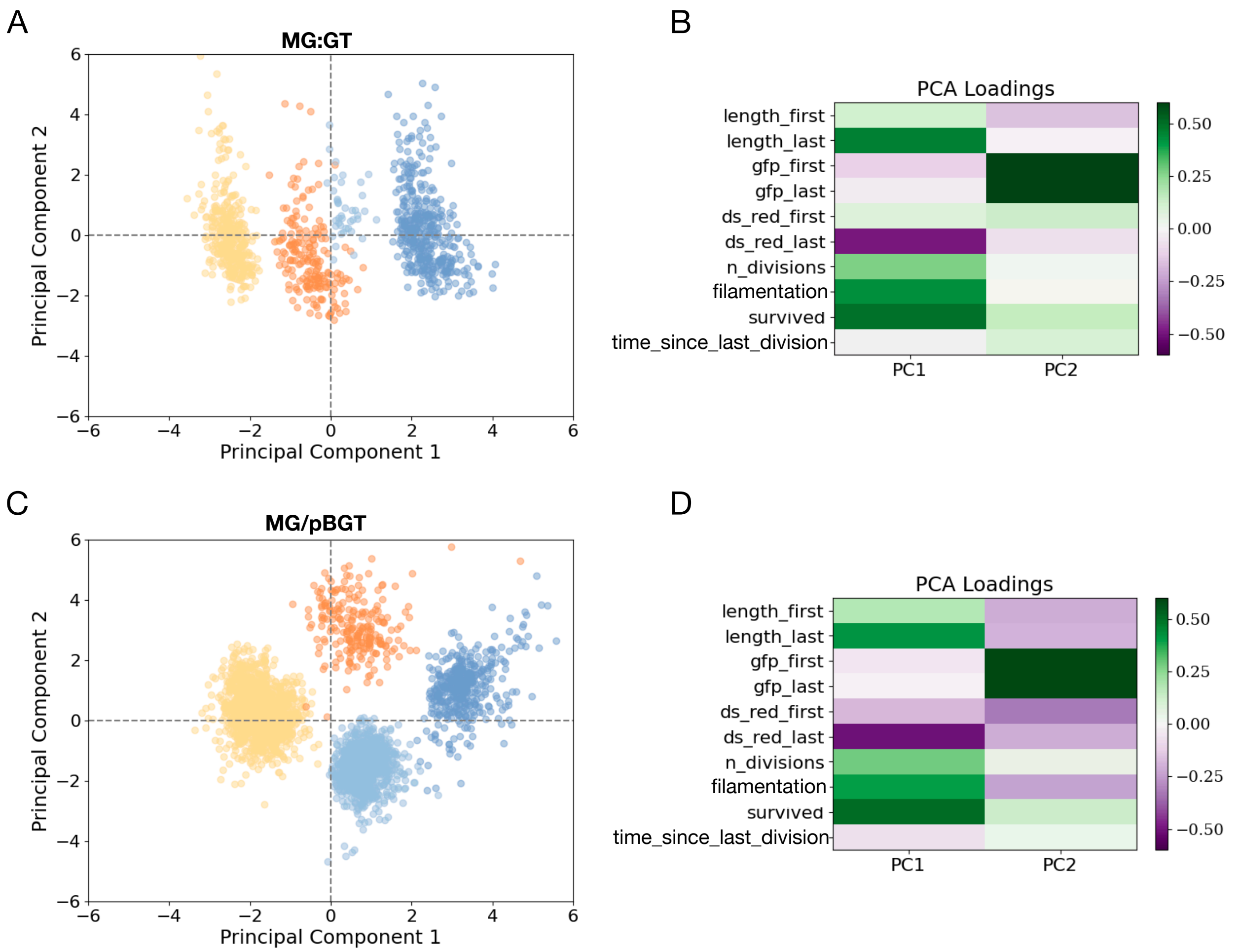

Figure S12. Principal Component Analysis shows the importance of cell length and GFP intensity in cell survival. A) The PCA plot shows the distribution of cells in the MG:GT population in a reduced dimensional space, where each point represents a sample and its position reflects its overall similarity to other samples based on multiple variables. The two axes of the plot correspond to the two principal components that explain the highest proportion of variance in the data. The color code indicates different subpopulations: cells that survived in blue and cells that were killed in orange. Dark colors represent cells that produced filaments and light colors cells where cell length was maintained. B) Individual contribution of each variable for the first two components of the PCA analysis. C) Dimensionality reduction analysis using PCA revealed distinct separation between surviving and antibiotic-killed cells, as well as between filamentous and non-filamentous cells, within the MG/pBGT population. D) PCA loadings associated with the MG:/pBGT population. For the first component, survival and the length of the cell explained most of the variability, while the second component was determined by the GFP intensity before and after drug exposure.

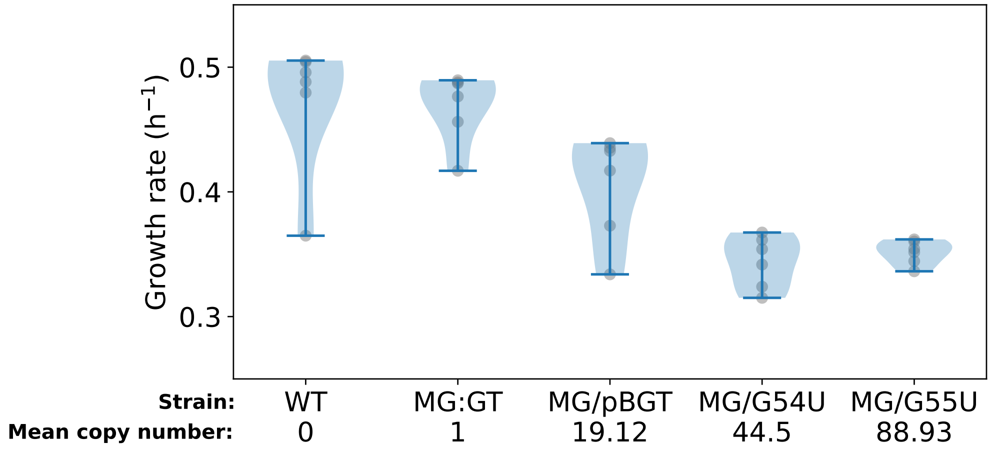

Figure S13. Fitness cost estimated at a population-level. Maximum growth rates estimated for different strains used in our study. There is a negative association between fitness in drug-free environments and the number of plasmids carried by each cell. A follow-up Tukey's Honest Significant Differences analysis yields the following strain pairs with p-value < 0.05 : WT-MG/pBGT, MG/pBGT-MG:GT, MG/pBGT-MG/G54U.

Strain ID	Mean PCN	AMP MIC ($\mu g/mL$)	Fitness (relative to MG)
MG	NA	4	1
MG:GT	NA	512	1.01
MG/pBGT	19.12 ± 1.53	$8,192$	0.943
MG/G54U	44.5 ± 3.81	$32,768$	0.793
MG/G55U	88.93 ± 15.65	$32,768$	0.557

Table S1. List of *Escherichia coli* MG1655 strains used in this study.

Parameter	Description	Value	source/rationale
μ	Mean plasmid copy number	19	Obtained from [1, **?**]
σ	Coefficient of variation max PCN	0.25	Value inferred from fluorescence data
Eff_i	Cell efficiency	1×10^6	This work *
V_{max}	Maximal uptake rate	2.5×10^{-8}	This work *
K_m	Half-saturation constant	0.25	This work *
p	Cost per plasmid	0.003	Table S1, $p = (1 - 0.943)/19$
ATP_{max}	Critical ATP concentration for division	1	This work *,**
δ	Antibiotic degradation	1×10^{-10}	Derived from [**?**]
B_0	Initial number of cells	1×10^3	1:1,000 bottle neck

Table S2. Parameter values employed in the numerical simulations of the agent-based model. * Values were adjusted based on the ODE model described in [**?**], modified to align with computational constraints, to maintain a maximum cell number, and to ensure cell divisions occur every 30 model iterations. ** Values set for ease of interpretation and visualization in the simulation results.

SI: AGENT-BASED MODEL OF PLASMID POPULATION DYNAMICS

Antibiotic resistance in bacteria is a pressing concern in modern medicine. A significant factor behind this resistance is the presence of plasmids— DNA molecules that replicate independently of the chromosome and can carry genes providing resistance to antibiotics. Understanding the dynamics of these plasmids—how they replicate, distribute, and interact with their bacterial hosts—can offer insights into the mechanisms that allow bacteria to survive antimicrobial treatment.

To provide a deeper understanding of these dynamics, especially in the context of fluctuating selection for plasmid-encoded genes, we designed a multi-level computational model (Figure SS14). This model integrates intracellular plasmid dynamics into an ecological framework, simulating key cellular processes with the aim of evaluating the role of copy number variability in the survival of bacterial populations. By recognizing the various factors contributing to PCN heterogeneity, the framework offers a controlled environment to study how PCN distributions determine bacterial survival under different antibiotic conditions.

Model overview

In this model, individual bacteria are simulated as computational agents, each with distinct properties and behaviors [2, 3], consuming a single limiting resource in a homogeneous environment and susceptible to the action of a bactericidal antibiotic.

- **Plasmid Dynamics**: In our model, we assume each cell has an average plasmid copy number (PCN) of approximately 19, based on [1]. To introduce inherent noise in PCN, cells are assigned a random maximal plasmid copy number. This inherent heterogeneity, arising from the varied maximal plasmid counts across cells, complements other sources of variability in plasmid dynamics, such as stochastic plasmid replication [4] and uneven plasmid segregation during cell division[5, 6]. We model plasmid replication as a saturating function to represent the self-limiting nature of plasmid replication.

- **Bacterial growth**: Growth is based on a limiting resource, R, following a Michaelis-Menten kinetics by an uptake function $U(R) = (V_{max} * R)/(K_m + R)$, this uptake is in turn transformed into energy with a given efficiency that is hindered by a per-cell plasmid cost. Cells accumulate energy until they surpass a threshold and undergo division. Upon division, the cell energy is noisily distributed between the divided cells and an energetic cost for division is applied.

- **Antibiotic-induced killing**: We use a linear approximation of the experimentally determined Minimum Inhibitory Concentration (MIC) of different strains. This allows us to derive a resistance/susceptibility profile for each cell based on its current plasmid count. In particular, we consider that the level of resistance is linear with respect to its PCN, as supported by our data (Figure 2D).

Key Assumptions and Considerations

- Bacterial growth and survival are influenced by both the internal state of each cell (e.g. the number of plasmid copies carried) and the external concentrations of resources and antibiotics.

- The model introduces variability in plasmid distributions within cells and incorporates randomness in both the probability of plasmid replication and in the segregation of plasmids during cell division. In cases of high-copy plasmids, like the ones used in this study, replicative noise emerges as intracellular selection favors overreplication. This selective pressure relieves the need for precise copy number control within cells [7].

- During division, plasmid segregation between mother and daughter cells is considered a random process, with each plasmid having an equal chance of being in either cell. We assume there is no active partitioning or post-segregational killing mechanisms [8].

- We consider that, while plasmids provide with antibiotic resistance to their hosts, they are also associated with a fitness cost [9, 10].

Numerical implementation

The agent-based model was developed using the Object-Oriented Programming (OOP) paradigm and implemented in the Julia programming language (Version 1.8.4).

- **Key Libraries/Dependencies:** We used `Plots.jl` for visualization, `DataFrames.jl` for data manipulation, and other essential Julia libraries to facilitate the simulation and analysis.
- **Repository Access:** The complete code, along with any associated documentation, is available in a public repository at https://github.com/ccg-esb-lab/pBGT.

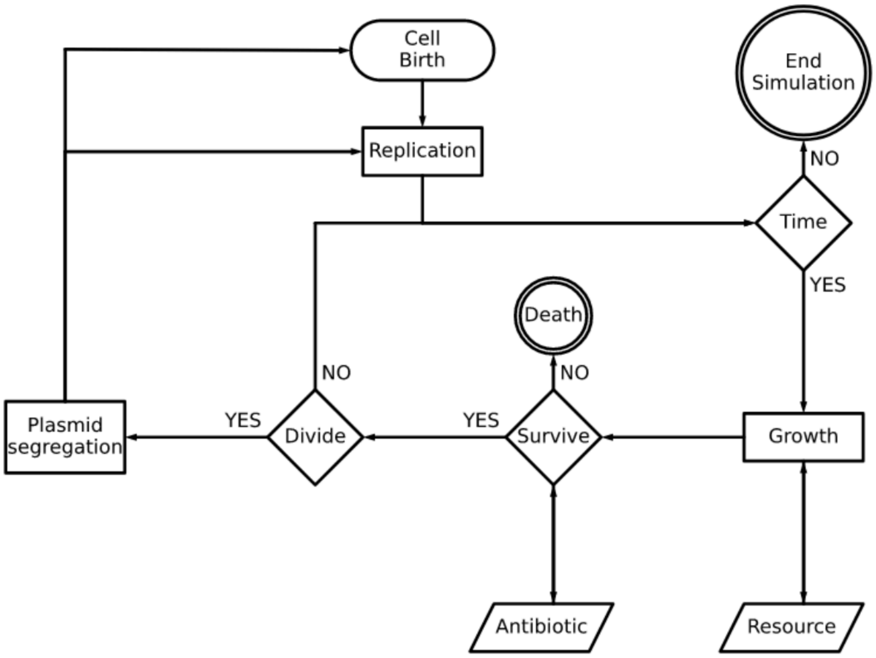

Figure S14. Model diagram illustrating the life cycle of a bacterial cell in our agent-based model. Beginning with **Cell Birth**, a new bacterial cell is introduced into the system. Following birth, cells consume resources and accumulate ATP. Simultaneously, the plasmids initiate their **Replication** process within this cell, where they multiply based on specific probability functions that depend on the current PCN. Upon reaching a certain ATP threshold, the cell undergoes **Division**, resulting in two daughter cells. A critical stage during this division is the **Plasmid Segregation**, where the replicated plasmids are randomly distributed between the two offspring cells, each having an equal chance of inheriting any particular plasmid. At every iteration, a **Survival Check** assesses each cell's viability against the action of antibiotics. Cells that pass this check continue to **Grow** and re-embark on the cycle, whereas those that fail are marked for **Death** and removed from the system. This iterative process encapsulates the dynamic interplay of bacterial growth and plasmid dynamics.

1 MODELING BACTERIAL GROWTH

Resource-limited growth

The growth of bacterial cells is defined using a Michaelis-Menten equation [11]. In the context of our model, this function is used to characterize how bacterial cells use a limiting resource (R) for their growth. As cells deplete the resource, the rate of resource uptake can change, as illustrated in Figure SS15 and described by:

$$U(R) = \frac{V_{max} * R}{K_m + R}.$$

ATP is the primary energy currency of cells. The efficiency of converting resources to ATP, thus, directly impacts the cell's energy availability and growth rate. Therefore our model considers that resources are then converted to 'ATP' with a specific efficiency. Parameters V_{max} and K_m have been parameterized to achieve division, in average, every 30 time-steps (so the model time span would be in minutes). We consider that all bacterial cells share uniform values for parameters V_{max} and K_m. Also, it is assumed that the external environment is well-mixed and constant, providing optimal conditions for bacterial growth.

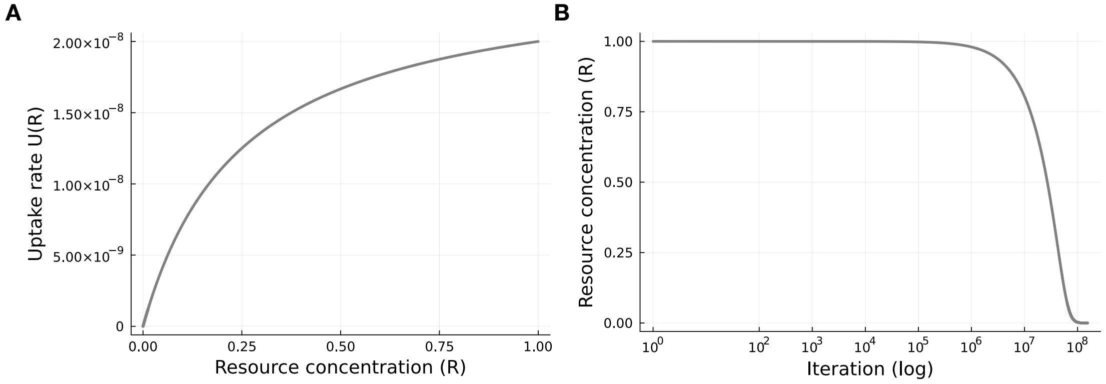

Figure S15. A) Relationship between uptake rate and resource concentration (R), illustrating how the rate changes in response to varying levels of available resources. B) Change in resource concentration over time, showing a decreasing trend as resources are progressively consumed.

Fitness cost associated with plasmid-bearing

We define the cell's ATP production efficiency, denoted as Eff_i, as a coefficient that quantifies the energy expenditure associated with maintaining and replicating plasmids within the cell. Therefore, the instantaneous ATP increment for a cell is:

$$ATP_i = Eff_i * Pcost_{total} * U(R)$$

with the fitness burden associated with plasmid-bearing defined as:

$$Pcost_{total} = 1 - \mu_i(t) * Pcost_{per-plasmid}.$$

where $\mu_i(t)$ is the copy number of cell i at time t (see section 3 for more details) and $Pcost_{per-plasmid}$ is a parameter representing the cost per plasmid. This value is calculated by dividing the total fitness cost, as identified in experiments, by the average number of plasmids in a cell [1]. Figure S3 illustrates the dynamic interplay between plasmid copy number and ATP concentration within individual cells and their progeny.

Cell Division

In our model, cell division is intricately tied to ATP dynamics. As ATP accumulates within a cell, reaching a certain threshold signifies that there is enough energy and metabolic activity to warrant division. When a cell divides, the energy is not split evenly: daughter cells inherit ATP based on a normal distribution, specifically Normal$(0.5, 0.15)$. This introduces variability in the initial energy content of these newly formed cells, mimicking the natural variability observed in biological systems. Additionally, the division process itself has an inherent cost. The mother cell experiences a 5% reduction in its ATP content after division. While this may briefly hinder the cell's metabolic activity, it provides an approximation of the energy costs associated with division.

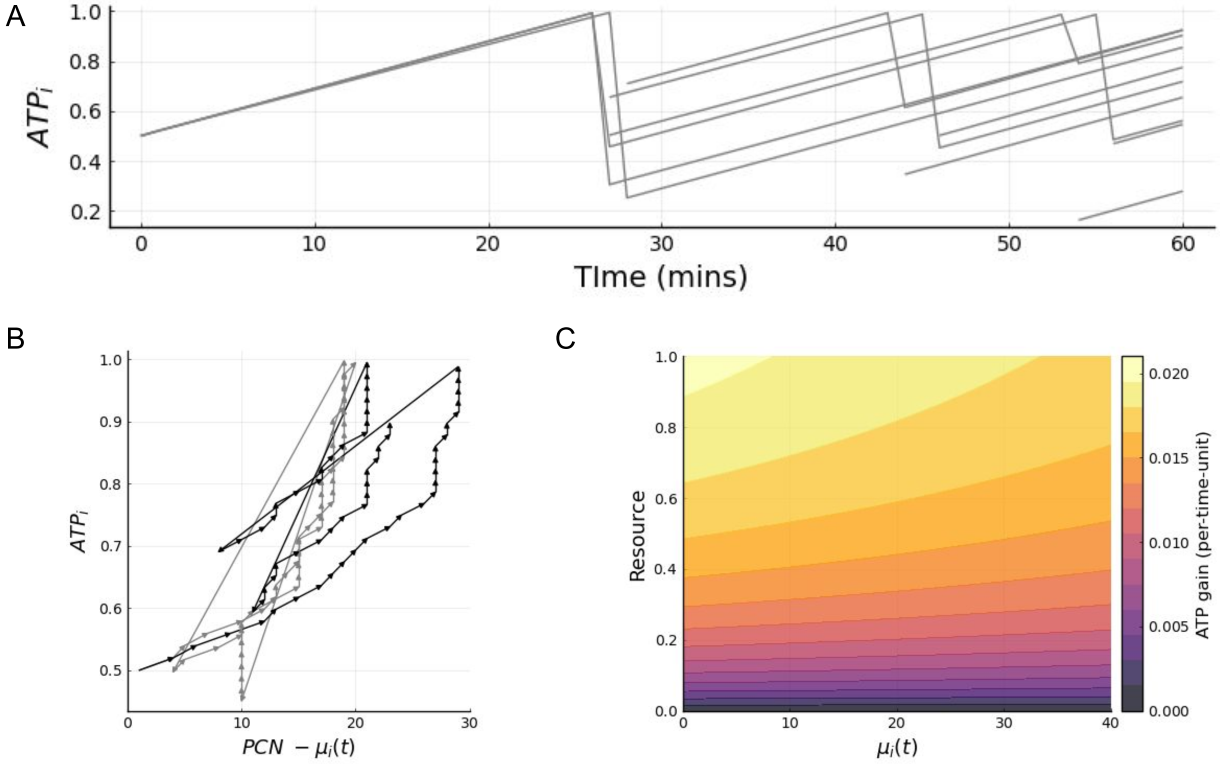

Figure S16. A) Shows the growth dynamics of three initial cells and their progeny. Two cells begin with identical conditions, a maximum plasmid copy number ($\hat{\mu}_i$) of 19 and an initial plasmid copy number ($\mu_i(t)$) of 1, while the third starts with a $\hat{\mu}_i$ of 30 and a $\mu_i(t)$ of 1. Note the slower growth phase for the cell with a $\hat{\mu}_i$ of 30, and subsequent growth desynchronization due to variable replication rates and uneven ATP distribution. B) Depicts ATP-$\mu_i(t)$ phase trajectories for two initial cells from part A, illustrating non-linear increases in $\mu_i(t)$ and their adaptation following cell division. C) Provides an ATP dynamics overview with a color gradient indicating the amount of ATP accumulated per time unit by a single cell, as affected by resource availability and plasmid copy number. Contour lines highlight variations in instantaneous ATP uptake, reflecting the fitness costs of plasmid carriage.

2 BACTERIAL POPULATION DYNAMICS

Our model of bacterial population dynamics focuses on how individual bacterial cells grow over time in response to available resources. Each bacterial cell competes for these resources in every discrete time-step of the simulation, converting them into ATP, which is vital for growth and division. Once a bacterial cell accumulates sufficient ATP, it undergoes division and creates a new individual.

2.1 Numerical experiment: Growth of a bacterial population

In our simulation experiment, we monitored both the status of each bacterial cell and the available resources throughout the process (Fig SS17).

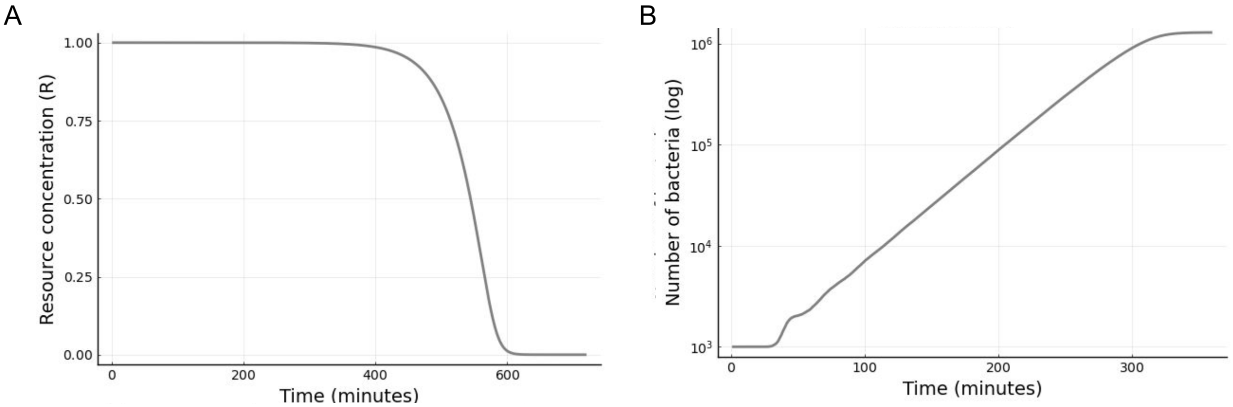

Figure S17. A) Resource concentration over time, illustrating the depletion of nutrients as they are consumed by the bacterial population. B) Number of bacterial cells over time, displayed on a log10 scale to effectively demonstrate the lag and exponential phases of growth, followed by stabilization as the population approaches carrying capacity due to resource depletion.

3 PLASMID DYNAMICS

3.1 Plasmid copy number control

Our model assumes that the control over plasmid copy number is not stringently regulated [12]. This variability is mathematically captured using an *a priori* population plasmid distribution modelled as a Normal(μ,σ), where μ symbolizes the average plasmid copy number and σ typifies the inherent variability. As the simulation initiates, each bacterial cell is assigned a maximal copy number, denoted $\hat{\mu}_i$, derived from the $N(\mu,\sigma)$. Alongside, a dynamic, time-dependent plasmid copy number, $\mu_i(t)$, represents the actual number of plasmids a cell possesses at any time instance t. Following a cell division event, the plasmid copy number undergoes segregation, after which each cell is assigned a new $\hat{\mu}_i$ for the forthcoming cycle.

3.2 Plasmids segregation

We consider that every plasmid within a cell holds the same chance of either translocating to the daughter cell or remaining with the mother, each event manifesting with a probability of 0.5. Upon successful segregation, a new maximal plasmid copy number, $\hat{\mu}_i$, is designated to both mother and offspring, drawn afresh from the overarching population distribution.

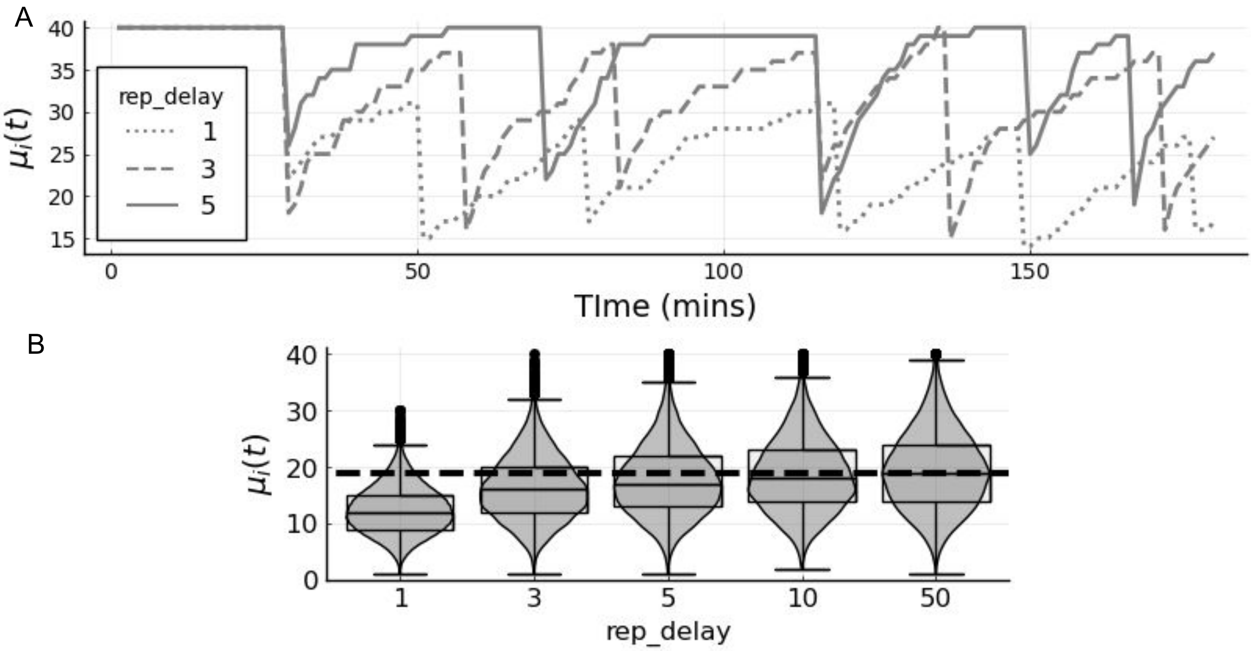

Figure S18. A) Plasmid replication dynamics of three cells with a mean plasmid copy number of 40, assuming no initial variation in PCN between cells. Replication delays are indicated by different line styles: dotted for $rep\_delay = 1$, dashed for $rep\_delay = 3$, and solid for $rep\_delay = 5$. A decline in PCN is noted with smaller rep_delay values, suggesting that plasmids may become unstable in the population without sufficient replication delay. B) Shows the impact of varying replication delay on the average PCN at the population level, with a mean of 19, as indicated by the thick horizontal dashed line. It is observed that lower rep_delay values are associated with a reduction in the average PCN, whereas higher values simulate the effect of near-instantaneous replication.

3.3 Plasmid replication

The process of plasmid replication within bacterial cells is modeled as a dynamic and stochastic process. In our model, we consider that the plasmid copy number within each cell, denoted as $\mu_i(t)$, increases towards a notional maximum value, $\hat{\mu}_i$, beyond which replication is increasingly less likely. The model does not employ a strict upper limit on the copy number but instead uses a probabilistic approach to govern the likelihood of replication events. At each time point in the simulation, the probability of a plasmid being replicated is assessed. This probability, P_{rep}, is described by an asymptotically decreasing function of the current plasmid copy number, which can be expressed as

$$P_{rep} = 1 - \frac{\mu_i(t)}{\hat{\mu}_i}.$$

As the number of plasmids within a cell approaches $\hat{\mu}_i$, the rate of replication slows, simulating the self-regulation observed in actual biological systems where the efficiency of replication decreases as certain intracellular resource limits are approached. This model is based from existing models in the literature [3] that use sigmoidal or other nonlinear functions to capture the complex interplay between plasmid concentration and replication probability. By adopting an asymptotic approach, our model allows us to simulate the naturally occurring feedback mechanisms that cells employ to balance plasmid load while avoiding the imposition of arbitrary hard thresholds.

We introduce a replication delay parameter, rep_delay, in our model to simulate the temporal separation between bacterial cell division and plasmid replication. Figure S5 highlights the impact of varying the rep_delay parameter, illustrating how replication timing influences plasmid stability and distribution within bacterial populations. To understand the implications of this parameter, consider a hypothetical bacterial

population where the average PCN per cell is $\mu = 19$. In this model, cells divide every 30 iterations, and the PCN within a cell is effectively halved at each division. For example, a cell with a PCN of 100 at the time of division would reduce to 50 immediately afterwards. Assuming each plasmid can replicate once per iteration, the PCN could increase to 80 before the next division. Subsequently, following each division, the PCN would decrease and then rise again, but with a diminishing peak due to the replication delay – 40 rising to 70, then 35, and so forth. This pattern illustrates how replication delay can regulate the accumulation of plasmids within a cell, affecting both the stability and distribution of plasmids across the population.

4 ANTIBIOTIC DYNAMICS IN BACTERIAL POPULATIONS

The dynamics of antibiotic resistance and susceptibility are governed by the complex interaction between bacterial cells, plasmids and antibiotic molecules. In our model, a bacterium's resistance to antibiotics is associated with its plasmid copy number (PCN), which is assumed to correlate with beta-lactamase production and, consequently, the level of resistance it exhibits. This relationship is expressed as:

$$S_i = \frac{A}{r_i} \text{ where } r_i = m\mu_i(t) + b.$$

Here, A denotes the antibiotic's environmental concentration. The term $\mu_i(t)$ represents the present PCN of the i-th bacterial cell, while m and b are coefficients from the experimentally derived linear relationship between the MIC and PCN. We consider that if $S_i < 1 + \text{Kill-noise}$, with Kill-noise being a value randomly drawn from a Normal(0,0.1) distribution, the bacterium remains viable; if not, it is eliminated (Figure SS19).

In our study, we assume a linear relationship between PCN and antibiotic resistance. This assumption is particularly relevant in systems where gene dosage correlates with PCN, such as in our system where the plasmids encode an antibiotic-degrading enzyme. For instance, a higher number of plasmid copies results in increased production of beta-lactamase, enhancing bacterial resistance to beta-lactam antibiotics. While this assumption is supported by empirical observations, it is based on data from a limited dataset, due to the experimental challenges in controlling PCN.

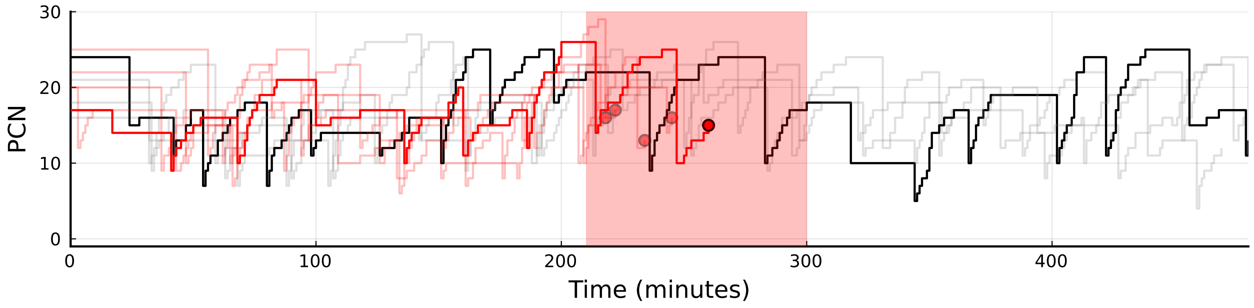

Figure S19. Intracellular plasmid dynamics for individual cells. The lines represent the PCN of each cell, with changes in PCN resulting from two random processes: plasmids replicate during cell growth and segregate between daughter cells during cell division. The red area denotes the interval of drug exposure, and cells that were killed are marked in red lines with a dot showing the moment of death. Cells that survived drug treatment and continued to segregate and replicate plasmids are depicted in gray, an illustrative trajectory is presented with a black line.

5 NUMERICAL EXAMPLE: DOSE-RESPONSE EXPERIMENT

To explore the interaction between bacterial populations and antibiotics, we conducted a dose-response experiment using our agent-based model (Figure SS20). In this experiment, we systematically varied the antibiotic concentration and observed its impact on population dynamics, measured as a reduction in the cell numbers. The Inhibitory Concentration (IC) is defined as the normalized drug concentration at which a specified level of reduction in bacterial cell numbers, as measured at the end of the experiment, is observed in our simulations.

We initialized the simulation with a heterogeneous bacterial population and exposed it to a range of antibiotic concentrations. For each concentration, we tracked the growth and survival of individual bacterial cells over a defined time period. The antibiotic concentration was represented as a parameter in our model, allowing us to simulate its effect on bacterial growth.

As the simulation progressed, we monitored key population metrics such as cell density, plasmid distribution, and overall fitness under different antibiotic concentrations. By analyzing these metrics, we gained insights into how the population's response changed as antibiotic levels increased or decreased.

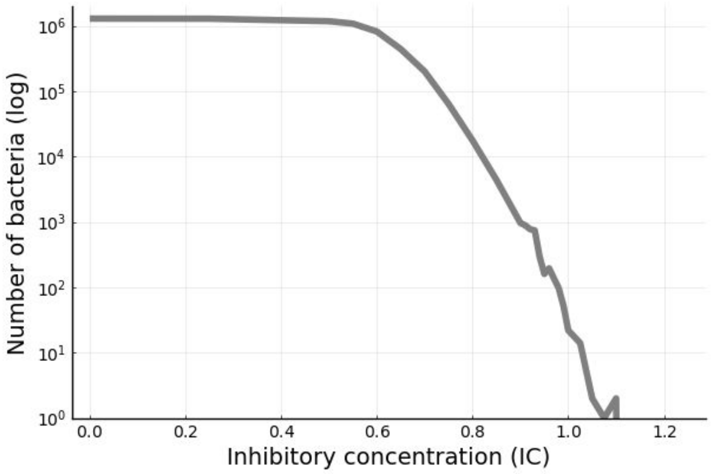

Figure S20. Dose-response experiment performed using the agent based model illustrates the relationship between antibiotic concentration (in units of MIC) and the number of bacterial cells (in $\log_{10}$ scale). As the antibiotic concentration increases, there is a corresponding reduction in the number of cells, reflecting the antibiotic's inhibitory effect on the bacterial population.

6 NUMERICAL EXAMPLE: SURVIVAL ASSAY

Throughout this study, we have consider a mean PCN of $\mu = 19$, based on empirical data from experimental observations (Figure 2D). This specific mean is representative of bacterial populations under study conditions. We also chose a 25% coefficient of variation (CoV) to reflect the natural variability in PCN among individual bacterial cells. This level of variation is crucial for comprehending antibiotic resistance dynamics, influencing the survival and adaptability of bacterial populations under diverse environmental stresses, as illustrated in Figure 1C.

In our our simulations, we explored various μ_{max} values, revealing a linear correlation between PCN and MIC. Figure SS21 highlights the effect of different CoV values on survival probabilities under a constant selective pressure (the MIC of a population with $\sigma^2 = 0$ and mean $\mu_{max} = 19$). A lower CoV indicates a more uniform distribution of PCN within a population, which may lead to more consistent patterns of survival or resistance. In contrast, a higher CoV introduces a greater diversity in PCN, yielding a wider spectrum of survival outcomes. This variability underscores the significant role of genetic variability in shaping the resilience and adaptability of bacterial populations when exposed to antibiotic stress.

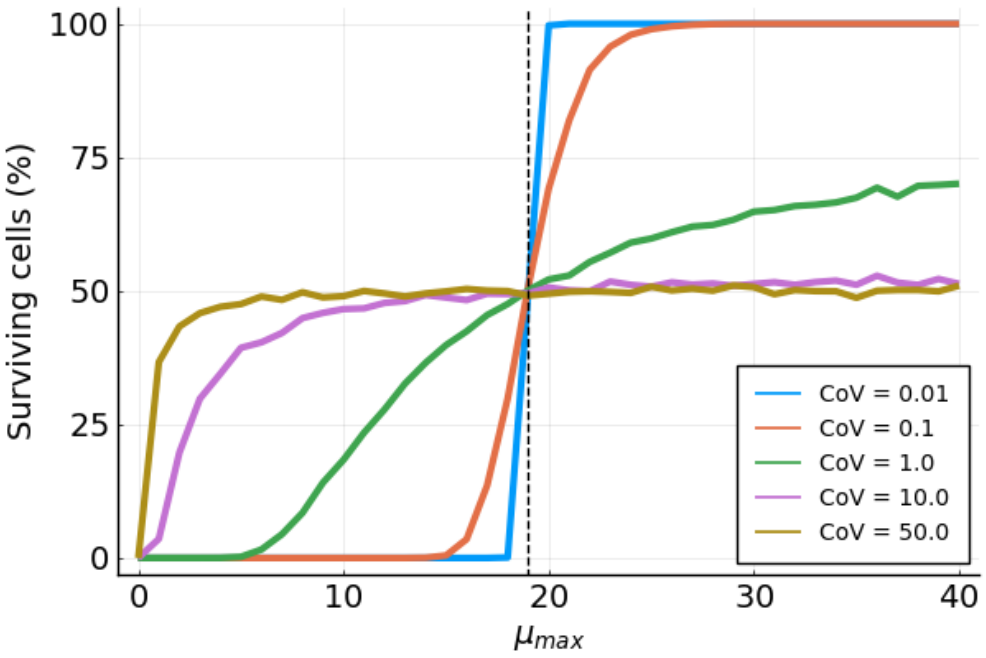

Figure S21. Percentage of surviving cells in experiments with an antibiotic concentration corresponding to the MIC of a population with a fixed PCN variance ($\sigma^2 = 0$) and a mean PCN of $\mu_{max} = 19$. The CoVs are represented by different colors: blue for CoV=0.01, orange for CoV=0.1, green for CoV=1.0, purple for CoV=10.0, and brown for CoV=50.0. The data illustrates that lower CoVs correspond to a more distinct threshold effect in survival rates, resembling a step function, while higher CoVs lead to a more uniform survival probability across the range, indicative of increased PCN diversity within the population.

7 NUMERICAL EXAMPLE: SERIAL TRANSFER EXPERIMENT

Serial transfer experiments are a widely utilized method in microbiology to study microbial evolution over successive generations [13]. In such experiments, microbial populations are grown for a fixed time interval, after which a small fraction is transferred to fresh media to start a new growth cycle. This procedure is repeated over several cycles, allowing for the observation of evolutionary changes over many generations in a relatively short period of time (Figure SS22).

In our modeling approach, we emulate this protocol through consecutive single-day growth simulations, tailored with day-specific antibiotic conditions. After each day's simulation, a representative sample of the surviving population is selected in line with the initial population size. To simulate the effects of nutrient scarcity-induced starvation – a stress period that cells undergo before transfer to fresh media – we reduce the intracellular ATP levels to 25% of their baseline values. This adjustment is informed by empirical observations that report a significant decline in metabolic activity and energy availability during starvation. This is illustrated in the simulation as a lag phase at the onset of the growth curve, reflecting the delay in population growth as cells adapt to the newly abundant resources following a period of scarcity.

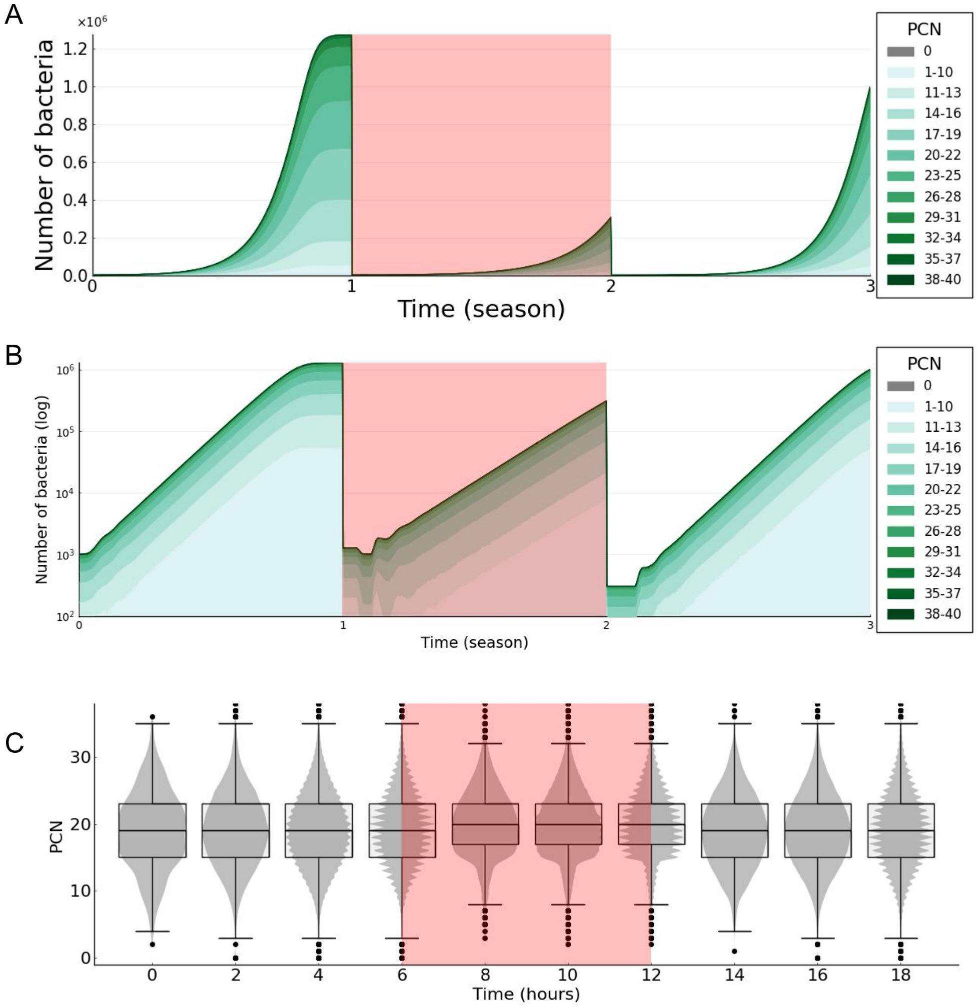

Figure S22. A) Computer simulations of the agent-based model showing the number of bacteria as a function of time in a three-season serial dilution experiment (with antibiotic deployed in season 2). Stacked areas represent the fraction of cells in the population with different PCNs (plasmid-free in grey and increasing PCNs in a range of green). B) This plot presents the same simulation data as in A) but displayed on a logarithmic scale to enhance the visibility of changes within populations having lower cell counts. C) Distribution of PCN at different time-points shows that the mean PCN of the population increases during exposure to antibiotics (area highlighted in red) but is restored after drug withdrawal.

8 ATP THRESHOLDS IN ANTIBIOTIC ACTION AND REPLICATION DYNAMICS

In our simulation, we connect the effectiveness of β-lactam antibiotics to the level of ATP within the cell. The parameter `antibiotic_action` reflects this connection and is set between 0.5 and 1.0, starting at 0.9 in our model. This number represents the ATP level at which antibiotics start to affect the cell. Cells divide when their ATP level, ATP_i, reaches 1. By setting `antibiotic_action` to 0.9, we ensure that the antibiotic starts to act before the cell divides, but not immediately after division when ATP levels are low. This setting helps us understand how antibiotics influence the distribution of plasmids in the bacterial population at different stages of cell growth.

The relationship between the time it takes for plasmids to replicate, known as replication delay, and the moment when antibiotics take effect is important in our model. This relationship is critical for young cells that have not yet reached the full ATP level needed for division. To explore how these two factors work together, we ran simulations that varied both the replication delay and the antibiotic action threshold. Our goal was to observe how these changes affect the plasmid numbers in bacteria when exposed to antibiotics. We carried out these simulations in a controlled way that eliminated the random effects of antibiotic killing and used a strong concentration of antibiotics to clearly see their impact (Figure SS23).

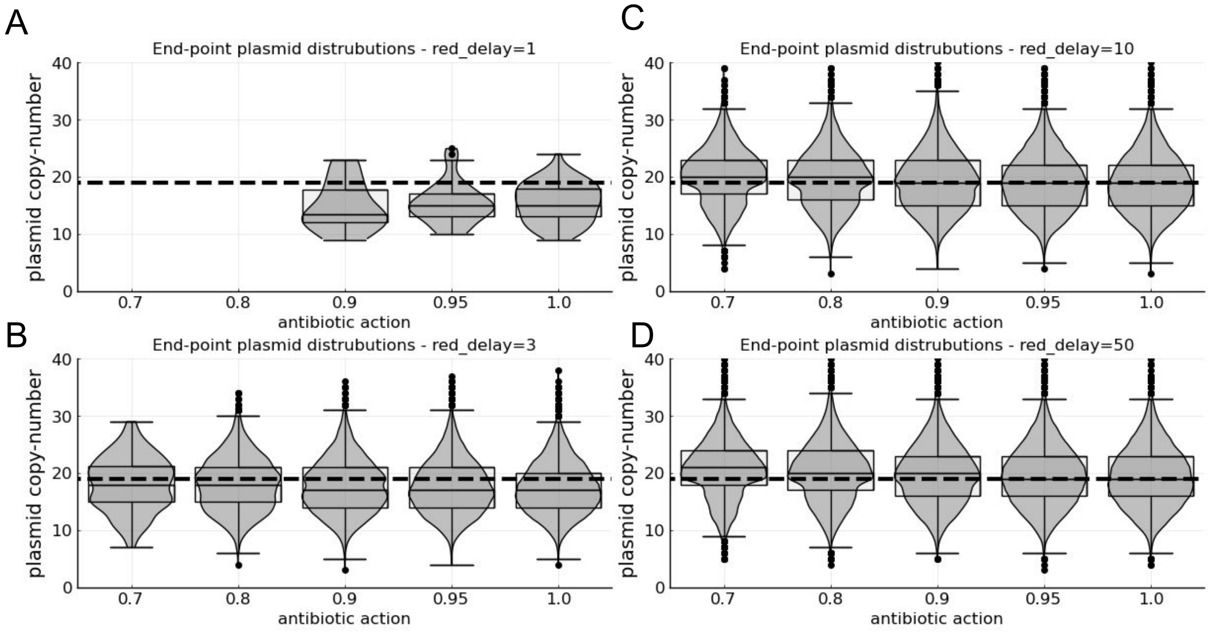

Figure S23. A) Illustrates the plasmid distribution in bacterial populations exposed to a range of antibiotic concentrations, with a replication delay set to 1. B) Shows the distribution when the replication delay is increased to 3. x C) Further extension of the replication delay to 10 accentuates the observed trends and highlights the consequent implications on bacterial populations. D) With the replication delay set at its maximum tested value, 50, the figure depicts the pronounced effects of an extended delay on plasmid distribution in the presence of antibiotics.

9 COMPARING DIFFERENT SOURCES OF NOISE IN THE PCN DISTRIBUTION

In our exploration of plasmid dynamics, we used the computational model to evaluate the impact of various noise sources on variations in PCN within bacterial cells. Figure SS24A illustrates the dynamics of plasmid replication over time with the initial PCN variability removed. The color gradient below the line at PCN 19 indicates the cells that have undergone division, each starting with varied PCNs due to segregation noise. These cells then engage in regulated plasmid replication, introducing another layer of variability. Eventually, as resources dwindle, most cells cease division, converging towards their maximum PCN, $\hat{\mu}_i$.

In Figure SS24B, we simulate instantaneous plasmid replication by setting a high *rep_delay*. The dispersion of PCNs around 10 is due to the cells having just divided, with the resulting variability arising from asymmetrical plasmid segregation at cell division. Figure SS24C depicts the impact of allowing replication noise while assuming perfect plasmid segregation. This scenario reveals a broader spread of PCNs over time, indicating increased variability due to replication noise alone.

In Figure SS24D, we examine a context devoid of external noise sources, focusing exclusively on intrinsic plasmid characteristics. A function ensuring equitable plasmid distribution during cell division was implemented, yielding a coefficient of variation of zero. Cells in this model showed minimal PCN variation, with their plasmid counts fixed at the maximum or halved after division.

Figure SS24E adds variability to the plasmid copy number control mechanism. The resulting PCN dynamics were markedly heterogeneous, with individual cell plasmid counts both below and above the population's mean PCN, highlighting the randomness of this noise source. Lastly, Figure SS24F integrates all identified sources of noise, including inconsistencies in plasmid replication, initial PCN variation, and division-related fluctuations. This examples reveals not only a population with a highly diverse PCN distribution but also underscores the transition from homogeneity to diversity in PCN as cells undergo division around time point 30. This transition highlights the stochastic nature of plasmid distribution and replication across cell generations, thereby explaining the observed variability in plasmid distribution as a result of the cumulative effect of multiple sources of noise.

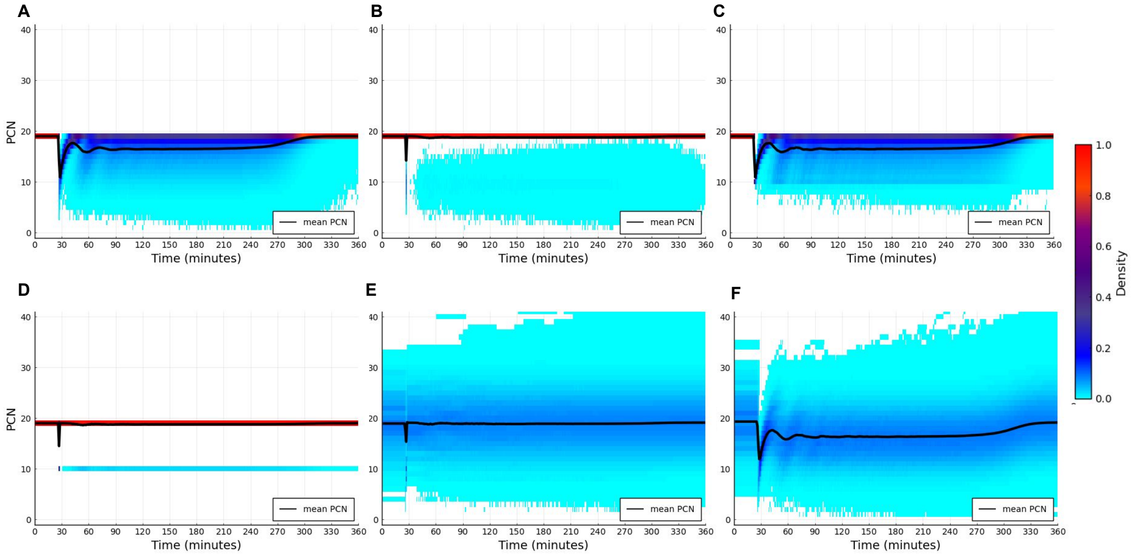

Figure S24. This figure illustrates the evolution of the PCN distribution in a bacterial population over time, assuming an initial state where all cells have identical PCN and ATP levels. The color gradient represents the frequency of cells with specific PCNs, ranging from cyan (low frequency) to red (high frequency), with black solid lines indicating the population's average PCN. A) Noise in plasmid dynamics without initial PCN variability. B) Plasmid dynamics with segregation noise as the sole source. C) Dynamics affected solely by replication noise. D) Plasmid dynamics without external noise factors. E) Dynamics with initial PCN variability incorporated. F) Comprehensive plasmid dynamics with all recognized noise sources, highlighting the shift from homogeneity to diversity in PCN as cells divide after time point 30, showcasing the stochastic nature of plasmid distribution and replication in cell generations.

10 EXPLORING DIFFERENT DISTRIBUTIONS IN PCN VARIABILITY

In our model, the initial distribution of PCN within the bacterial population is crucial. While we have primarily employed a Normal distribution to reduce biases, but we acknowledge the potential relevance of exploring alternative distributions. Therefore, we used the model to examine the effects of various PCN distributions on bacterial dynamics under antibiotic stress, including Uniform, Gamma, Beta (Left-leaning and Right-leaning), Biweight, SkewNormal, LogitNormal, and Normal.

Figure SS25A depicts theoretical PCN distribution within bacterial populations, contrasting conditions with antibiotic exposure (red) and without drugs (grey). The grey distribution signifies the baseline PCN in a drug-free environment. Upon introducing antibiotics, a selective pressure is applied, conferring a survival advantage to bacteria with specific PCN values. This leads to the red distribution, indicating a shift in the PCN landscape that favors bacteria with a higher plasmid count. The mean PCN for each scenario is annotated above the respective distributions.

Our simulations generally show a trend where antibiotic presence instigates an increase in mean PCN; this is indicative of selective pressure favoring higher plasmid counts. Notwithstanding, the magnitude of this shift in mean PCN is not uniform across different distributions. The interplay between the initial PCN distribution and antibiotic-induced selection pressure leads to diverse outcomes. In some cases, we observe a marked increase in mean PCN values, whereas in others, the increase is more subtle.

Moreover, in our model, variability within the PCN distributions correlates with enhanced survival rates in the presence of antibiotics, with more diverse populations demonstrating greater resistance (Figure SS25B). Nevertheless, this survival advantage is mitigated when the antibiotic concentration is decreased, especially in populations characterized by a narrower range of PCN variability.

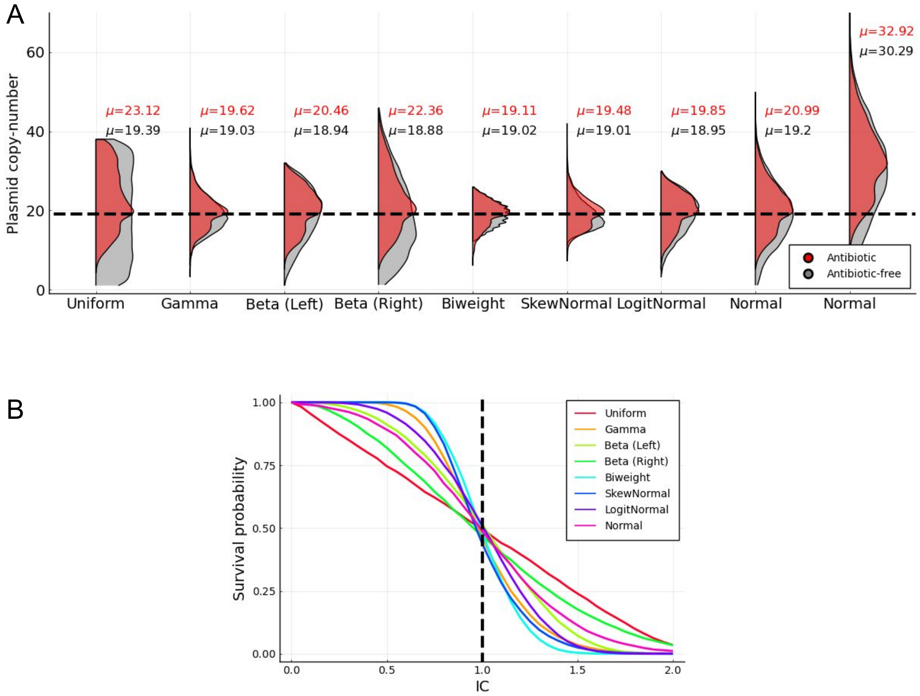

Figure S25. A) The figure displays the PCN distribution of bacterial populations when exposed to antibiotics (depicted in red) and in the absence of drugs (shown in grey). Note that antibiotic exposure results in most PCN distributions leaning towards higher values, indicating a heightened plasmid copy number in the presence of drugs. B) This panel portrays the survival probability of bacterial populations as it relates to selective pressure. Each plotted line corresponds to the estimated survival rates for the various PCN distributions analyzed. Across the board, in high-drug environments, survival rates surpass those expected from a uniform PCN distribution. However, in environments with lower drug concentrations, survival rates dip below those expected for a uniform PCN distribution.

REFERENCES

[1] San Millan, A., Escudero, J. A., Gifford, D. R., Mazel, D. & MacLean, R. C. Multicopy plasmids potentiate the evolution of antibiotic resistance in bacteria. *Nature ecology & evolution* **1**, 1–8 (2016).

[2] Kreft, J.-u. Mathematical modeling of plasmid dynamics. In *Molecular Life Sciences*, 1–6 (Springer, 2014).

[3] Hernández-Beltrán, J. C. R., San Millán, A., Fuentes-Hernández, A. & Peña-Miller, R. Mathematical models of plasmid population dynamics. *Frontiers in Microbiology* **12**, 606396 (2021).

[4] Jahn, M., Vorpahl, C., Hübschmann, T., Harms, H. & Müller, S. Copy number variability of expression plasmids determined by cell sorting and droplet digital pcr. *Microbial cell factories* **15**, 1–12 (2016).

[5] Novick, R. P. & Hoppensteadt, F. On plasmid incompatibility. *Plasmid* **1**, 421–434 (1978).

[6] Jahn, M., Günther, S. & Müller, S. Non-random distribution of macromolecules as driving forces for phenotypic variation. *Current opinion in microbiology* **25**, 49–55 (2015).

[7] Paulsson, J. Multileveled selection on plasmid replication. *Genetics* **161**, 1373–1384 (2002).

[8] Salje, J. Plasmid segregation: how to survive as an extra piece of dna. *Critical reviews in biochemistry and molecular biology* **45**, 296–317 (2010).

[9] San Millan, A. & MacLean, R. C. Fitness costs of plasmids: a limit to plasmid transmission. *Microbiology spectrum* **5**, 10–1128 (2017).

[10] Andersson, D. I., Patin, S. M., Nilsson, A. I. & Kugelberg, E. The biological cost of antibiotic resistance. *Enzyme-Mediated Resistance to Antibiotics: Mechanisms, Dissemination, and Prospects for Inhibition* 339–348 (2007).

[11] Kessick, M. The kinetics of bacterial growth. *Biotechnology and Bioengineering* **16**, 1545–1547 (1974).

[12] Del Solar, G. & Espinosa, M. Plasmid copy number control: an ever-growing story. *Molecular microbiology* **37**, 492–500 (2000).

[13] Buckling, A., Craig Maclean, R., Brockhurst, M. A. & Colegrave, N. The beagle in a bottle. *Nature* **457**, 824–829 (2009).
