# Supplementary material for: Plasmid-mediated phenotypic noise leads to transient antibiotic resistance in bacteria
Source: Nat Commun. 2024 Mar 23;15:2610. doi: 10.1038/s41467-024-45045-0 (PMC10960800; doi:10.1038/s41467-024-45045-0)
Supplement: Supplementary file 3 — Description of Additional Supplementary Files [file 41467_2024_45045_MOESM3_ESM.pdf]

Description of Additional Supplementary Files

Supplementary Movie 1: MG/pBGT exposed to a semi-lethal pulse of AMP

Supplementary Movie 2: MG:GT exposed to a semi-lethal pulse of AMP.
